# The Sossige

**Published:** 1895-07

**Authors:** 


**The Sossige.**—"The horse is a very useful animal," wrote Johnny in his composition, "but if I can't have my sossiges made out of pig's meet, I don't want no sossiges.—*The American.*

